# Supplementary material for: The Social Lives of Free-Ranging Cats
Source: Animals (Basel). 2022 Jan 5;12(1):126. doi: 10.3390/ani12010126 (PMC8749887; doi:10.3390/ani12010126)
Supplement: Supplementary file 1 [file animals-12-00126-s001.zip › animals-1465205-supplementary.pdf]

animals

MDPI

Table S1. Studies on FRC intraspecific social behavior. The location and social behavior(s) measured or observed for each study are provided. Behavioral categories are as follows: Affiliative (AFF), Agonistic (AGON), Reproductive (REPRO), Caregiving (CARE), Cooperative, (COOP), and Other (O).

Location	Paper	Social Behavior(s) Measured/Observed
Dockyard: Portsmouth, Hampshire, England	[24]	AFF: Allogroom, Tail Up, Nose sniff, Head-rub<br>AGON: Attack/Strike, Submit, Threaten, Stiffen & leave, Flee, Avoid, Leave, Chase, Threaten & Follow, Stare, Watch<br>REPRO: Waiting, Sexual Behavior (Mounting/Mating)
Dockyard: Avonmouth Docks, Bristol, England	[26]	AFF: Actual Contact (instances in which two cats were aware of each other's presence).<br>REPRO: Vocalizations (Calling)
Island: Ainoshima, Japan	[27]	REPRO: Copulation<br>AGON: Aggression, Submission
	[28]	REPRO: Copulation<br>AGON: Aggression
	[29]	REPRO: Distance (between an estrous female and courting males, at 1 min intervals), Male-Male Mounting (one male mounts another, biting the others neck. Does not consider intromission.)
	[30]	AFF: Affiliative Behavior AGON: Aggressive Behavior CARE: Social Behavior (between queen and her kittens) COOP: Caregiving Behavior (between an adult cat and kitten that is not her own offspring)
	[31]	REPRO: Mount Rejection (e.g., pawing, hissing, and rushing before and after mounting, but before copulation), Mount Acceptance (all observed mounting behaviors)
Park: Regent's Park, London, England	[32]	AFF: Allorub, Tail Up (greeting behaviors)<br>AGON: Aggressive Behavior (displays, paw slapping, and yowling)
City: Urban environment in Israel	[33]	AFF: Amicable Interactions<br>AGON: Aggressive Behavior
City: Residential neighborhood in city of Tel Aviv, Israel	[34]	AGON: Slapping, Chasing, Vocalizations (hiss, yowl and growl), Threat Displays (approach and stare), Physical Attacks
	[35]	Same as [34]
	[36]	AGON: Aggressive behaviors in [34], Submissive Behaviors (move away, run away, crouch), Results of Agonistic Interactions (one cat demonstrated an advantage by engaging in aggressive behavior- "winner", while the other cat showed submissive behavior- "loser").
City: Market square/City Center, "Piazza Vittorio Emanuele" in Rome, Italy	[37]	REPRO: Female Estrus (documented from behavioral patterns such as vocalizations, rubbing, rolling, crouching, acceptance of male copulation)<br>REPRO: Copulatory Behavior, Mounting Behavior (successful mounts are those with pelvic thrusting and intromission (M) and false mounts are those with pelvic thrusting and no intromission (MW)).
	[47]	REPRO: Courting Behavior, Mounting (scored for each male courting a female).<br>AGON: Male Agonistic Behavior. Included Threats (given/received by each male, e.g., paw striking, assuming a threatening posture, hissing, ear flattening), Ritualized Vocal Duels, Submissive Postures (in response to threats, e.g., couching, leaving, or fleeing), Copulation Interference, Outcomes of Agonistic Interactions (an individual cat was considered the "victor" of an agonistic interaction if they were the individual showing dominant postures while the other

		cat displayed submissive postures or left the vicinity.)
	[49]	REPRO: Courting Behavior (male following/running after female and alternating this with attempts to copulate), Female Acceptance (if female let the male mount), Female Rejection (if female behaved aggressively, causing physical separation), and Threats (rank order of behavior between dyads, e.g., paw striking, displaying a threatening posture, ear flattening).
City: Large Courtyard; "Garbatella" Rome, Italy	[38]	AGON: Aggressive Threats (paw striking, biting, threatening postures, pointing, staring at, baring canines), Chasing, Vocal Duels, Actual Fighting, Submissive Behaviors (crouching with flattened ears, avoiding, retreating, fleeing and hissing at), Dominance Rank (dominant cats are those that engaged in more aggressive behavior than was displayed to them OR received more submissive behavior than they displayed. Only behaviors that occurred in the absence of food were included and hissing was excluded), and Feeding Hierarchy (dyad ranking determined using the same methods as dominance however only behaviors within 1 m of the food were included).
	[39]	AFF: Allorub, Nose Sniff, Tail Up<br>AGON: As described in [38]
	[40]	AFF: Allogroom, Allorub, Nose Sniff, Passive Contact, Proximity (within 1 m)<br>AGON: As described in [38]
City: Fori di Traiano: Historical ruins, Centre of Rome, Italy	[42]	AGON: Aggressive Behavior (paw striking, displaying a threatening posture, chasing, ritualized vocalizations and physical contests), Submissive Behavior (including crouching with the ears flattened, avoiding, retreat, fleeing, hissing).<br>RANK: Outcomes of Agonistic Interactions, as in [47].
City: Croix Rousse Hospital Park, Center of Lyons, France	[48]	REPRO: Mounting (of females by males).
	[67]	AGON: Direction of Agonistic Interactions (between males as in [47]).
	[68]	AGON: Direction of Agonistic Interactions (males were classified into one of three social ranks, high, medium, or low. Ranks were formed by dividing all males into three equal groups so each rank had an equal number of cats).
City: Two sites in Italy (Fori di Traiano & Garbatella) and one in France (Croix Rousse)	[41]	AFF: Nose Sniff, Passive Contact, Allorub.<br>AGON: Aggressive Behaviors (threating, vocal duel, real duel, chasing), Submissive Behaviors (avoiding, leaving, crouching, flying, hissing).<br>REPRO: Mount Attempts, False Mounts, True Mounts, Social Rolls (display behavior)
City: Adachi Ward in Tokyo Metropolis, Japan	[16]	AGON: Pursuit of Cats<br>CARE: Nursing
Rural: Roland Laundre Farm, Green Bay, Wisconsin, USA	[43]	AFF: Alloub, Allogroom, Nose Touch (during greeting)<br>AGON: Antisocial Actions (such as striking at/growling)
Rural: Church Farm, Bradford, Devon, UK	[44]	AFF: Proximity-Seeking, Allogroom, Allorub, Social Play<br>AGON: Aggression (aggression sniff)<br>CARE/COOP: Parental or Alloparental Behavior (grooming, nursing, bringing prey to kittens)
Rural: Aimargues & Saint Just-Chaleyssin, village near Lyon, France	[45]	REPRO: Infanticidal Behavior (killing of kittens by adult male cats)
Waste Site: Oberon, New South Wales, Australia	[60]	O: Cat Interactions

Table S2. Studies on FRC interspecific social behavior. The location, species, and social behavior(s) measured or observed for each study are provided. Behavioral categories are as follows: Affiliative (AFF) and Other (O).

Location	Species	Paper	Social Behavior(s) Measured or Observed
University Campus: Nanjing University, Jiangsu Province, China	Human	[50]	AFF: Affiliative Socialization Score (calculated as the sum of all social behaviors shown toward the unfamiliar person. Each behavior's score is as follows: Approach (2), Proximity within 1 m (1), Stays within 1 m (1), Allows Petting (1), Allows holding (3), Meows at tester (3), Purrs (4), Reacts to toys (1), Plays with toys (2), Approaches and rubs on legs (2)).
Rural: Mental Health Center in Alma Michigan, USA	Human	[51]	AFF: Approaching People, Greeting People, and Initiating Physical Contact (e.g., tactile contact, sitting on the client's lap).
Island: Jekyll Island on Georgia's Southeastern coast	Wildlife	[52]	O: Watching, Pursuing, Approaching, Avoiding/Fleeing, Attacking, Fighting, Close Proximity during Eating & Drinking (or any other behavior unrelated to hunting).
